# Supplementary material for: Comparative Analysis of Carbohydrates, Nucleosides and Amino Acids in Different Parts of Trichosanthes kirilowii Maxim. by (Ultra) High-Performance Liquid Chromatography Coupled with Tandem Mass Spectrometry and Evaporative Light Scattering Detector Methods
Source: Molecules. 2019 Apr 11;24(7):1440. doi: 10.3390/molecules24071440 (PMC6479868; doi:10.3390/molecules24071440)
Supplement: Supplementary file 1 [file molecules-24-01440-s001.pdf]

Table S1. Contents (μg/g) of investigated compounds in nine parts of different cultivars of *T. kirilowii* (n=3)

Samples	Analytes (mg/g)														TN
	1	2	3	4	5	6	7	8	9	10	11	12	13	14	
**A(S1)**	0.0011	0.0005	0.1558	0.3852	0.0000	0.0000	0.0016	0.0005	0.0001	0.0019	0.0004	0.0001	0.0433	0.0001	0.5907
**A(S2)**	0.0008	0.0002	0.0738	0.2179	0.0000	0.0000	0.0004	0.0001	0.0001	0.0008	0.0002	0.0001	0.0527	0.0002	0.3472
**A(S3)**	0.0006	0.0002	0.1012	0.2093	0.0000	0.0000	0.0009	0.0002	0.0001	0.0012	0.0002	0.0001	0.0889	0.0005	0.4035
**A(S4)**	0.0008	0.0004	0.1074	0.3088	0.0000	0.0000	0.0005	0.0003	0.0001	0.0020	0.0004	0.0000	0.0755	0.0000	0.4960
**A(S5)**	0.0016	0.0008	0.1156	0.2934	0.0000	0.0000	0.0014	0.0002	0.0001	0.0018	0.0006	0.0000	0.1054	0.0002	0.5212
**A(S6)**	0.0018	0.0008	0.1210	0.3298	0.0000	0.0000	0.0002	0.0004	0.0001	0.0020	0.0006	0.0002	0.1473	0.0008	0.6051
**A(S7)**	0.0016	0.0005	0.1634	0.4264	0.0000	0.0000	0.0017	0.0008	0.0001	0.0018	0.0006	0.0001	0.0430	0.0002	0.6402
**B(S1)**	0.0013	0.0004	0.1772	0.5111	0.0000	0.0000	0.0037	0.0002	0.0001	0.0022	0.0004	0.0002	0.0875	0.0001	0.7843
**B(S2)**	0.0012	0.0005	0.1381	0.2168	0.0000	0.0000	0.0028	0.0001	0.0001	0.0023	0.0003	0.0001	0.0758	0.0002	0.4384
**B(S3)**	0.0008	0.0003	0.1237	0.2772	0.0000	0.0000	0.0021	0.0000	0.0001	0.0045	0.0002	0.0000	0.1225	0.0000	0.5314
**B(S4)**	0.0012	0.0004	0.1604	0.3750	0.0000	0.0000	0.0045	0.0002	0.0001	0.0027	0.0005	0.0001	0.1137	0.0001	0.6590
**B(S5)**	0.0017	0.0008	0.1659	0.3559	0.0000	0.0000	0.0043	0.0001	0.0001	0.0027	0.0005	0.0000	0.0853	0.0000	0.6172
**B(S6)**	0.0008	0.0004	0.1362	0.2974	0.0000	0.0000	0.0005	0.0001	0.0001	0.0014	0.0002	0.0001	0.1860	0.0004	0.6235
**B(S7)**	0.0024	0.0010	0.1953	0.5526	0.0000	0.0000	0.0035	0.0009	0.0001	0.0045	0.0009	0.0001	0.0557	0.0002	0.8171
**C(S1)**	0.0145	0.0065	0.2343	0.9227	0.0000	0.0000	0.0053	0.0016	0.0002	0.0097	0.0059	0.0001	0.2180	0.0000	1.4188
**C(S2)**	0.0157	0.0062	0.1774	0.7284	0.0000	0.0001	0.0105	0.0022	0.0009	0.0079	0.0063	0.0001	0.1954	0.0000	1.1511
**C(S3)**	0.0076	0.0037	0.1968	0.6865	0.0000	0.0000	0.0062	0.0015	0.0002	0.0088	0.0037	0.0000	0.2047	0.0000	1.1197
**C(S4)**	0.0124	0.0051	0.1938	0.9264	0.0000	0.0000	0.0135	0.0017	0.0002	0.0171	0.0060	0.0000	0.1938	0.0000	1.3700
**C(S5)**	0.0147	0.0055	0.2006	0.6471	0.0000	0.0000	0.0087	0.0012	0.0002	0.0096	0.0055	0.0000	0.2063	0.0000	1.0994
**C(S6)**	0.0131	0.0051	0.2297	0.7212	0.0000	0.0000	0.0042	0.0010	0.0001	0.0088	0.0054	0.0000	0.2410	0.0001	1.2298
**C(S7)**	0.0120	0.0048	0.2428	1.0342	0.0000	0.0000	0.0063	0.0073	0.0004	0.0101	0.0064	0.0000	0.0730	0.0000	1.3974
**D(S1)**	0.0024	0.0012	0.1639	0.2537	0.0000	0.0000	0.0220	0.0008	0.0001	0.0175	0.0005	0.0001	0.0570	0.0043	0.5235
**D(S2)**	0.0010	0.0004	0.1359	0.1607	0.0000	0.0000	0.0041	0.0000	0.0000	0.0020	0.0003	0.0000	0.0396	0.0019	0.3460

**D(S3)**	0.0005	0.0003	0.1237	0.1160	0.0000	0.0000	0.0032	0.0000	0.0001	0.0023	0.0002	0.0000	0.1315	0.0036	0.3813
**D(S4)**	0.0021	0.0009	0.1442	0.4035	0.0000	0.0000	0.0028	0.0002	0.0002	0.0076	0.0008	0.0000	0.0555	0.0003	0.6182
**D(S5)**	0.0032	0.0013	0.1445	0.3150	0.0000	0.0000	0.0034	0.0002	0.0001	0.0077	0.0008	0.0000	0.0301	0.0012	0.5075
**D(S6)**	0.0010	0.0005	0.1256	0.1542	0.0000	0.0000	0.0011	0.0002	0.0000	0.0077	0.0003	0.0000	0.1229	0.0036	0.4171
**D(S7)**	0.0021	0.0009	0.1708	0.3117	0.0000	0.0000	0.0026	0.0001	0.0001	0.0049	0.0008	0.0001	0.0729	0.0010	0.5679
**E(S1)**	0.0771	0.0267	0.0645	0.8951	0.0000	0.0345	0.0318	0.7311	0.0002	0.0293	0.0176	0.0001	0.0949	0.0001	2.0030
**E(S2)**	0.0805	0.0257	0.0265	0.3750	0.0000	0.0284	0.0264	0.3564	0.0002	0.0094	0.0178	0.0012	0.0720	0.0002	1.0198
**E(S3)**	0.0710	0.0210	0.0383	0.3647	0.0004	0.0149	0.0198	0.3891	0.0003	0.0162	0.0193	0.0006	0.1444	0.0004	1.1005
**E(S4)**	0.0656	0.0238	0.1360	0.4682	0.0503	0.0041	0.0662	0.3038	0.0007	0.0607	0.0314	0.0002	0.0027	0.0000	1.2136
**E(S5)**	0.0776	0.0281	0.0953	0.1072	0.0000	0.0025	0.1659	0.0249	0.0034	0.0039	0.0117	0.0001	0.0237	0.0000	0.5443
**E(S6)**	0.0734	0.0279	0.0962	0.3054	0.0003	0.0036	0.1009	0.0644	0.0006	0.0078	0.0177	0.0008	0.0187	0.0001	0.7178
**E(S7)**	0.0787	0.0266	0.0030	0.2502	0.0000	0.0274	0.0618	0.1779	0.0006	0.0583	0.0303	0.0020	0.0598	0.0000	0.7766
**F(S1)**	0.1113	0.0416	0.2287	0.8942	0.0001	0.0120	0.0243	0.2340	0.0003	0.0311	0.0634	0.0013	0.1398	0.0002	1.7823
**F(S2)**	0.1007	0.0321	0.1293	0.5440	0.0000	0.0017	0.0187	0.0642	0.0002	0.0087	0.0581	0.0010	0.1729	0.0001	1.1318
**F(S3)**	0.0919	0.0285	0.1383	0.5903	0.0001	0.0058	0.0246	0.1812	0.0002	0.0170	0.0668	0.0009	0.1829	0.0002	1.3287
**F(S4)**	0.0782	0.0265	0.0453	0.6197	0.0007	0.0118	0.0386	0.3887	0.0002	0.0173	0.0565	0.0023	0.0829	0.0002	1.3688
**F(S5)**	0.0757	0.0239	0.0072	0.4306	0.0000	0.0012	0.0339	0.1226	0.0003	0.0082	0.0560	0.0003	0.2109	0.0001	0.9708
**F(S6)**	0.1094	0.0355	0.1730	0.6725	0.0003	0.0090	0.0043	0.2453	0.0003	0.0182	0.1212	0.0060	0.1722	0.0001	1.5671
**F(S7)**	0.0893	0.0264	0.0657	0.6017	0.0000	0.0031	0.0115	0.0368	0.0002	0.0099	0.0693	0.0033	0.1157	0.0000	1.0330
**G(S1)**	0.0068	0.0023	0.1539	0.4508	0.0031	0.0000	0.0012	0.0036	0.0001	0.0010	0.0040	0.0001	0.0422	0.0000	0.6689
**G(S2)**	0.0083	0.0025	0.0484	0.1717	0.0765	0.0000	0.0005	0.0014	0.0000	0.0005	0.0032	0.0000	0.0396	0.0000	0.3527
**G(S3)**	0.0160	0.0037	0.2361	0.4509	0.0023	0.0003	0.0033	0.0050	0.0003	0.0020	0.0060	0.0016	0.0980	0.0001	0.8256
**G(S4)**	0.0187	0.0044	0.1950	0.4509	0.0824	0.0000	0.0004	0.0032	0.0001	0.0017	0.0129	0.0001	0.0517	0.0000	0.8216
**G(S5)**	0.0216	0.0063	0.1585	0.4564	0.0000	0.0072	0.0081	0.2584	0.0001	0.0042	0.0083	0.0013	0.0125	0.0000	0.9428
**G(S6)**	0.0041	0.0017	0.0096	0.0710	0.0030	0.0001	0.0001	0.0188	0.0000	0.0019	0.0029	0.0000	0.0027	0.0002	0.1161
**G(S7)**	0.0035	0.0012	0.0323	0.1082	0.0620	0.0000	0.0000	0.0043	0.0001	0.0008	0.0016	0.0000	0.0292	0.0001	0.2433

**H(S1)**	0.0010	0.0004	0.0187	0.0398	0.0000	0.0000	0.0000	0.0036	0.0001	0.0011	0.0007	0.0001	0.0033	0.0000	0.0683
**H(S2)**	0.0000	0.0000	0.0016	0.0001	0.0000	0.0000	0.0004	0.0005	0.0000	0.0098	0.0001	0.0000	0.0000	0.0000	0.0126
**H(S3)**	0.0000	0.0000	0.0019	0.0000	0.0000	0.0000	0.0006	0.0000	0.0001	0.0042	0.0000	0.0000	0.0424	0.0000	0.0491
**H(S4)**	0.0016	0.0009	0.0135	0.0569	0.0055	0.0000	0.0010	0.0077	0.0001	0.0023	0.0013	0.0010	0.0029	0.0000	0.0947
**H(S5)**	0.0000	0.0000	0.0003	0.0001	0.0000	0.0000	0.0003	0.0014	0.0000	0.0093	0.0001	0.0000	0.0219	0.0000	0.0334
**H(S6)**	0.0033	0.0011	0.0043	0.0682	0.0000	0.0007	0.0058	0.0507	0.0000	0.0005	0.0013	0.0000	0.0175	0.0000	0.1533
**H(S7)**	0.0000	0.0000	0.0047	0.0214	0.0000	0.0000	0.0045	0.0089	0.0000	0.0014	0.0001	0.0000	0.0065	0.0000	0.0282
**I(S1)**	0.0000	0.0000	0.0007	0.0000	0.0000	0.0000	0.0003	0.0000	0.0001	0.0142	0.0000	0.0000	0.0000	0.0000	0.0152
**I(S2)**	0.0002	0.0000	0.0003	0.0000	0.0000	0.0000	0.0027	0.0000	0.0000	0.0043	0.0000	0.0000	0.0000	0.0000	0.0077
**I(S3)**	0.0001	0.0000	0.0001	0.0001	0.0000	0.0000	0.0000	0.0000	0.0001	0.0001	0.0000	0.0000	0.0000	0.0000	0.0005
**I(S4)**	0.0000	0.0000	0.0019	0.0000	0.0000	0.0000	0.0026	0.0000	0.0000	0.0080	0.0000	0.0000	0.0000	0.0000	0.0125
**I(S5)**	0.0000	0.0000	0.0006	0.0000	0.0000	0.0000	0.0000	0.0000	0.0001	0.0074	0.0000	0.0000	0.0000	0.0000	0.0082
**I(S6)**	0.0000	0.0000	0.0019	0.0003	0.0000	0.0000	0.0093	0.0000	0.0002	0.0756	0.0000	0.0000	0.0000	0.0000	0.0873
**I(S7)**	0.0000	0.0000	0.0004	0.0000	0.0000	0.0000	0.0006	0.0000	0.0001	0.0088	0.0000	0.0000	0.0000	0.0003	0.0102

Continued of Table S1

**Samples**	**Analytes (mg/g)**													
	**15**	**16**	**17**	**18**	**19**	**20**	**21**	**22**	**23**	**24**	**25**	**26**	**27**	**28**
**A(S1)**	0.0478	0.0795	0.0163	0.0714	0.7770	0.0493	0.1345	0.0998	0.0611	0.0000	0.0001	0.0000	0.0801	0.5797
**A(S2)**	0.0331	0.1480	0.0713	0.1440	0.8657	0.1027	0.2065	0.0859	0.0897	0.0000	0.0001	0.0000	0.1054	0.2689
**A(S3)**	0.0080	0.0316	0.0132	0.0380	0.6733	0.0291	0.0875	0.0452	0.0308	0.1100	0.0001	0.0001	0.1891	0.5030
**A(S4)**	0.0194	0.0657	0.0175	0.0662	0.6385	0.0644	0.1172	0.1174	0.0505	0.1297	0.0001	0.0001	0.0476	0.4965
**A(S5)**	0.0877	0.2443	0.1030	0.2966	0.7778	0.0627	0.3124	0.0738	0.1421	0.1184	0.0001	0.0003	0.1303	0.8256
**A(S6)**	0.0912	0.2536	0.1231	0.2502	0.8022	0.1424	0.3144	0.1784	0.1970	0.0000	0.0001	0.0017	0.2502	0.5247
**A(S7)**	0.0739	0.3769	0.1618	0.3637	0.7320	0.1735	0.4081	0.1898	0.1546	0.1465	0.0001	0.0003	0.1878	0.9075
**B(S1)**	0.0390	0.1069	0.0225	0.0897	0.9047	0.0627	0.1673	0.1076	0.0644	0.1087	0.0001	0.0002	0.0890	0.5736
**B(S2)**	0.0627	0.3389	0.1178	0.3216	1.0093	0.1401	0.4140	0.1064	0.1807	0.1209	0.0001	0.0001	0.1256	0.3373

**B(S3)**	0.0189	0.0522	0.0140	0.0575	0.9014	0.0247	0.1250	0.0910	0.0528	0.1419	0.0001	0.0000	0.0671	0.4507
**B(S4)**	0.0475	0.3033	0.0882	0.2449	0.8303	0.1278	0.2828	0.1044	0.1021	0.0000	0.0001	0.0003	0.1173	0.6240
**B(S5)**	0.0945	0.2710	0.1303	0.2946	1.0451	0.0680	0.3241	0.1315	0.1945	0.0000	0.0001	0.0001	0.1387	1.0761
**B(S6)**	0.0294	0.1854	0.1453	0.1815	0.8067	0.0962	0.1506	0.2300	0.1186	0.1122	0.0001	0.0003	0.0648	0.4946
**B(S7)**	0.0857	0.5506	0.1979	0.4972	0.9277	0.1943	0.5736	0.1803	0.2182	0.1495	0.0001	0.0005	0.1716	1.3427
**C(S1)**	0.0487	0.1120	0.0154	0.1170	1.1132	0.0677	0.2452	0.2082	0.1523	0.1203	0.0001	0.0002	0.1406	0.4576
**C(S2)**	0.0720	0.2641	0.0689	0.3321	1.1141	0.1314	0.4787	0.1836	0.2475	0.1289	0.0001	0.0002	0.3296	0.2365
**C(S3)**	0.0301	0.0640	0.0100	0.0782	0.8482	0.0397	0.1571	0.1362	0.1128	0.1890	0.0001	0.0001	0.3780	0.3659
**C(S4)**	0.0451	0.1592	0.0261	0.1656	0.9999	0.1199	0.2435	0.1427	0.1411	0.1153	0.0001	0.0002	0.1453	0.4279
**C(S5)**	0.0667	0.2049	0.0532	0.2712	1.1084	0.0759	0.3606	0.2382	0.2070	0.1508	0.0001	0.0001	0.2968	0.8749
**C(S6)**	0.0411	0.1565	0.0613	0.1897	1.0475	0.1149	0.2234	0.3239	0.1584	0.1106	0.0001	0.0001	0.1001	0.4295
**C(S7)**	0.0628	0.2760	0.0689	0.3353	0.9288	0.1312	0.4575	0.2622	0.2013	0.2034	0.0001	0.0002	0.2780	0.8407
**D(S1)**	0.0431	0.0903	0.0198	0.0886	1.1435	0.0510	0.2161	0.1645	0.0616	0.1147	0.0001	0.0000	0.3034	0.3069
**D(S2)**	0.0518	0.2020	0.0930	0.1895	1.1578	0.1363	0.3450	0.1720	0.1246	0.0908	0.0001	0.0000	0.3535	0.2381
**D(S3)**	0.0143	0.0424	0.0162	0.0498	0.9967	0.0331	0.1197	0.0948	0.0434	0.1095	0.0001	0.0000	0.2421	0.3610
**D(S4)**	0.0317	0.0925	0.0203	0.0932	0.9091	0.0466	0.1824	0.1786	0.0665	0.0000	0.0001	0.0002	0.0729	0.3692
**D(S5)**	0.0720	0.1774	0.0785	0.2356	0.9748	0.0546	0.3269	0.1629	0.1555	0.1502	0.0003	0.0001	0.2338	0.7078
**D(S6)**	0.0576	0.2003	0.1334	0.2056	0.9369	0.1482	0.2889	0.1162	0.0998	0.0000	0.0001	0.0001	0.2026	0.2737
**D(S7)**	0.0756	0.3737	0.1581	0.3581	1.0616	0.1527	0.5035	0.2668	0.1594	0.0000	0.0001	0.0001	0.2316	0.6706
**E(S1)**	0.0417	0.1024	0.0478	0.1538	0.9443	0.0498	0.3754	2.1155	0.0006	0.2081	0.0003	0.0006	0.4113	1.7830
**E(S2)**	0.0360	0.1017	0.0516	0.1562	0.3988	0.0354	0.2845	1.5609	0.0012	0.1565	0.0003	0.0004	0.3133	0.8404
**E(S3)**	0.0188	0.0543	0.0369	0.0858	0.3434	0.0256	0.2095	1.2848	0.0157	0.1513	0.0004	0.0001	0.6169	0.9416
**E(S4)**	0.0559	0.1281	0.0598	0.1576	0.5045	0.0527	0.3374	1.8751	0.0002	0.2011	0.0004	0.0012	0.7295	1.2335
**E(S5)**	0.0515	0.1246	0.0708	0.1712	0.3014	0.0511	0.3770	0.4415	0.0028	0.2301	0.0007	0.0005	1.2793	0.5717
**E(S6)**	0.0366	0.0996	0.0478	0.1414	0.2242	0.0373	0.3002	0.8475	0.0007	0.2044	0.0005	0.0010	0.8333	1.0754
**E(S7)**	0.0493	0.1188	0.0457	0.1622	0.3056	0.0516	0.3296	0.8374	0.0138	0.1295	0.0002	0.0005	0.6140	0.9309

**F(S1)**	0.0995	0.2374	0.0513	0.3285	0.5644	0.1035	0.7567	0.3989	0.0014	0.3286	0.0001	0.0002	0.7870	1.4806
**F(S2)**	0.1359	0.3066	0.0663	0.4191	0.2538	0.1303	0.7118	0.3298	0.0012	0.2666	0.0002	0.0014	0.7082	0.8131
**F(S3)**	0.0566	0.1458	0.0331	0.2272	0.2691	0.0612	0.5086	0.2221	0.0002	0.2689	0.0002	0.0001	0.7578	0.5417
**F(S4)**	0.1462	0.3317	0.0723	0.3823	0.3546	0.1389	0.7515	0.3465	0.0845	0.2792	0.0001	0.0002	0.9601	0.5643
**F(S5)**	0.1829	0.4081	0.0842	0.5483	0.1550	0.1655	0.9761	0.4502	0.0005	0.3587	0.0002	0.0003	1.0568	0.7468
**F(S6)**	0.1244	0.2938	0.0899	0.4257	0.2163	0.1029	0.8264	0.2658	0.0225	0.1738	0.0002	0.0011	0.7266	1.5967
**F(S7)**	0.1704	0.3762	0.0792	0.4631	0.4108	0.1719	0.8847	0.4760	0.0272	0.3402	0.0002	0.0041	0.7100	0.9406
**G(S1)**	0.1172	0.2301	0.0422	0.2456	0.6271	0.1356	0.3927	0.3214	0.5553	0.1794	0.0001	0.1626	0.0243	1.1878
**G(S2)**	0.0870	0.1707	0.0326	0.2140	0.3479	0.0807	0.2591	0.2895	0.2987	0.0875	0.0001	0.0927	0.0213	0.2993
**G(S3)**	0.0266	0.0618	0.0118	0.1004	0.1516	0.0731	0.2379	0.1711	0.2267	0.1038	0.0002	0.1347	0.0700	0.4742
**G(S4)**	0.1004	0.2040	0.0472	0.2703	0.5371	0.1259	0.4449	0.3316	0.5311	0.1603	0.0001	0.1992	0.0174	0.9875
**G(S5)**	0.0807	0.1361	0.0392	0.2136	0.3825	0.1088	0.3961	0.2757	0.5161	0.2261	0.0002	0.1965	0.0609	1.2783
**G(S6)**	0.0671	0.1280	0.0319	0.1428	0.3152	0.0544	0.1944	0.1280	0.2051	0.0102	0.0001	0.0812	0.0575	0.3269
**G(S7)**	0.0325	0.0726	0.0125	0.0857	0.1341	0.0395	0.1312	0.1009	0.1222	0.0000	0.0000	0.0620	0.0220	0.1927
**H(S1)**	0.1077	0.2033	0.0444	0.2087	0.1645	0.0634	0.2728	0.1986	0.4377	0.1176	0.0000	0.0499	0.1908	0.1289
**H(S2)**	0.1834	0.3232	0.0650	0.3586	0.2796	0.0938	0.4053	0.4641	0.5517	0.1991	0.0001	0.0796	0.3929	0.2904
**H(S3)**	0.1131	0.2077	0.0440	0.2444	0.1984	0.0671	0.3284	0.2713	0.6128	0.0000	0.0000	0.0664	0.3364	0.0594
**H(S4)**	0.1731	0.3140	0.0600	0.3161	0.3494	0.0744	0.3915	0.3785	0.3026	0.2711	0.0001	0.1857	0.2730	0.4588
**H(S5)**	0.1855	0.3107	0.0741	0.3502	0.2619	0.0975	0.4303	0.3729	0.4941	0.2170	0.0001	0.0154	0.5000	0.3798
**H(S6)**	0.1004	0.1782	0.0396	0.1743	0.1401	0.0447	0.1843	0.1690	0.0682	0.0950	0.0000	0.0004	0.1859	0.1442
**H(S7)**	0.1467	0.2685	0.0532	0.2647	0.2934	0.0731	0.3161	0.2336	0.3648	0.1254	0.0000	0.1738	0.2871	0.1667
**I(S1)**	0.0656	0.1102	0.0313	0.1105	0.0870	0.0443	0.1478	0.1085	0.1008	0.0000	0.0000	0.0678	0.4317	0.0675
**I(S2)**	0.0289	0.0380	0.0196	0.0409	0.0194	0.0211	0.0599	0.0440	0.0471	0.0138	0.0001	0.0268	0.2271	0.0101
**I(S3)**	0.0183	0.0106	0.0078	0.0126	0.0238	0.0093	0.0213	0.0089	0.0257	0.0000	0.0000	0.0130	0.1102	0.0035
**I(S4)**	0.0507	0.0736	0.0273	0.0742	0.0498	0.0326	0.0965	0.0693	0.0781	0.0000	0.0000	0.0467	0.3293	0.0396
**I(S5)**	0.0430	0.0647	0.0226	0.0654	0.0230	0.0313	0.0916	0.0704	0.0715	0.0603	0.0000	0.0447	0.2821	0.0378

**I(S6)**	0.0989	0.1745	0.0628	0.1850	0.2310	0.0909	0.3189	0.2752	0.1899	0.0781	0.0001	0.0977	1.4216	0.2227
**I(S7)**	0.0488	0.0713	0.0230	0.0697	0.0329	0.0336	0.1026	0.0872	0.0792	0.0287	0.0001	0.0327	0.3327	0.0017

Continued of Table S1

Samples	Analytes (mg/g)								Analytes (%)				
	**29**	**30**	**31**	**32**	**33**	**34**	**35**	**TA**	**fructose**	**glucose**	**Raffinose**	**Stachyose**	**polysaccharide**
**A(S1)**	0.0049	0.0558	10.6274	2.2795	0.4363	0.1633	0.0021	15.5658	2.60	2.75	0.49	4.43	10.32
**A(S2)**	0.0069	0.0389	6.4090	1.3540	0.2862	0.0891	0.0010	10.3065	2.47	2.42	0.48	2.59	11.68
**A(S3)**	0.0063	0.0466	9.3230	2.0571	0.3467	0.1688	0.0011	13.7086	1.72	1.68	0.55	3.41	8.29
**A(S4)**	0.0048	0.0515	11.9702	2.7302	0.4332	0.1691	0.0013	17.1910	2.45	2.17	0.39	5.11	6.86
**A(S5)**	0.0109	0.1121	10.9478	2.1693	0.5941	0.3348	0.0015	17.3455	2.43	2.62	0.57	4.26	18.32
**A(S6)**	0.0458	0.1132	6.7538	1.7936	0.9075	0.1011	0.0009	12.8452	2.56	2.53	0.53	2.29	4.70
**A(S7)**	0.0182	0.0714	8.7233	2.0442	0.5235	0.2345	0.0007	15.4922	3.21	3.43	0.30	2.96	19.94
**B(S1)**	0.0124	0.0519	11.9511	2.2221	0.4477	0.1735	0.0022	17.1972	3.19	3.31	0.45	4.18	10.07
**B(S2)**	0.0394	0.0514	7.7168	1.2826	0.4236	0.1865	0.0013	12.9773	3.61	3.80	0.24	2.63	11.66
**B(S3)**	0.0051	0.0624	9.7075	2.1238	0.4142	0.3259	0.0015	14.6377	2.57	2.54	0.33	3.42	10.30
**B(S4)**	0.0206	0.0555	11.6337	2.5756	0.5427	0.2263	0.0008	17.9282	2.77	2.91	0.28	4.46	8.93
**B(S5)**	0.0070	0.1205	11.6809	3.0028	0.7123	0.4212	0.0025	19.7156	2.99	3.15	0.32	4.23	10.62
**B(S6)**	0.0270	0.0431	8.4565	1.5220	0.5199	0.1596	0.0005	13.3441	3.38	3.42	0.41	3.11	8.57
**B(S7)**	0.0213	0.0981	9.1033	2.1422	0.5541	0.2639	0.0009	17.2736	3.91	4.38	0.13	3.17	7.09
**C(S1)**	0.0098	0.0944	13.4546	1.9512	0.5863	0.1670	0.0018	19.0635	4.26	4.34	0.30	4.60	11.28
**C(S2)**	0.0097	0.1238	8.4996	1.3640	0.5250	0.1762	0.0013	14.2873	4.41	4.73	0.18	3.47	9.90
**C(S3)**	0.0053	0.0837	10.6343	1.7728	0.4570	0.1298	0.0008	15.4932	4.03	4.19	0.25	3.95	10.46
**C(S4)**	0.0108	0.0964	14.9663	2.7903	0.4603	0.2334	0.0010	21.2904	3.54	3.78	0.25	6.03	12.59
**C(S5)**	0.0047	0.1631	13.5687	2.2846	0.6665	0.5755	0.0014	21.1731	3.72	4.00	0.18	5.29	12.58
**C(S6)**	0.0103	0.0867	10.0054	1.4301	0.5275	0.1588	0.0007	15.1768	4.60	4.63	0.26	3.75	11.69
**C(S7)**	0.0100	0.1477	9.0850	1.9497	0.6139	0.2005	0.0020	16.0552	4.38	4.52	0.17	2.17	10.90

**D(S1)**	0.0020	0.0366	11.6316	2.3546	0.4737	0.2297	0.0020	17.3338	3.39	4.15	0.26	7.39	5.40
**D(S2)**	0.0007	0.0691	7.2842	1.4114	0.3882	0.1574	0.0032	12.4687	3.72	4.56	0.00	2.21	12.56
**D(S3)**	0.0017	0.0634	9.3873	2.0199	0.3659	0.1968	0.0020	14.1601	2.84	3.32	0.00	3.89	16.49
**D(S4)**	0.0084	0.0698	11.8503	3.0839	0.4736	0.2067	0.0020	17.7579	3.17	3.93	0.00	7.52	10.53
**D(S5)**	0.0034	0.1498	10.4661	2.1546	0.5422	0.2575	0.0035	16.9077	3.08	3.68	0.00	5.43	20.10
**D(S6)**	0.0052	0.0519	7.6387	1.4983	0.6689	0.1634	0.0008	12.6905	3.07	3.63	0.00	2.60	16.77
**D(S7)**	0.0045	0.0939	8.7173	1.9557	0.5503	0.2435	0.0013	15.5783	3.97	4.82	0.00	3.33	10.81
**E(S1)**	0.0227	0.1103	2.6686	0.5243	0.5180	0.0689	0.0055	10.1529	3.81	3.42	0.10	0.66	5.15
**E(S2)**	0.0249	0.0117	1.8092	0.2580	0.3753	0.0466	0.0016	6.4645	4.12	3.69	0.00	0.94	4.20
**E(S3)**	0.0083	0.0047	1.4771	0.2420	0.2603	0.0434	0.0047	5.8259	1.70	1.47	0.00	0.95	3.78
**E(S4)**	0.0420	0.0119	3.3677	0.6581	0.4856	0.0875	0.0049	9.9947	3.17	2.72	0.00	1.17	3.75
**E(S5)**	0.0247	0.0000	1.6808	0.6703	0.5191	0.1172	0.0060	6.6922	0.76	0.98	0.00	1.00	3.88
**E(S6)**	0.0150	0.0016	0.6569	0.3109	0.4171	0.0462	0.0049	5.3027	1.01	1.07	0.00	0.32	3.69
**E(S7)**	0.0173	0.0042	1.2099	0.4083	0.4829	0.0452	0.0067	5.7636	1.61	1.61	0.00	0.45	3.96
**F(S1)**	0.0062	0.1776	2.6671	0.7082	0.6701	0.0703	0.0067	9.4444	3.67	4.22	1.80	1.29	3.03
**F(S2)**	0.0283	0.0789	0.7067	0.4138	0.7466	0.0811	0.0042	6.2040	4.01	4.58	1.59	0.92	3.14
**F(S3)**	0.0029	0.1182	0.7614	0.2598	0.4861	0.0382	0.0028	4.7621	2.17	2.75	1.64	1.10	3.43
**F(S4)**	0.0024	0.1391	0.7577	0.5134	0.7926	0.0446	0.0033	6.6655	2.55	3.05	1.49	0.98	3.30
**F(S5)**	0.0057	0.0258	0.6810	0.6025	0.9223	0.0729	0.0040	7.4479	7.87	7.58	0.80	0.34	4.04
**F(S6)**	0.0110	0.2488	0.6867	0.4319	0.6490	0.0534	0.0034	6.9503	2.44	2.91	1.01	0.86	3.77
**F(S7)**	0.0709	0.2968	0.5596	0.5029	1.0036	0.0368	0.0032	7.5281	4.96	5.53	0.94	0.36	4.86
**G(S1)**	0.3536	0.2099	6.4685	1.5880	0.7280	0.0518	0.0028	13.6240	20.76	23.65	0.00	2.29	9.85
**G(S2)**	0.2086	0.0921	3.8111	0.6266	0.4394	0.0229	0.0053	7.4873	26.08	29.10	0.00	2.08	5.89
**G(S3)**	0.2326	0.1258	2.2687	0.6450	0.5694	0.0253	0.0010	5.7117	10.91	9.62	0.00	0.58	6.14
**G(S4)**	0.3885	0.2213	3.8389	0.9154	0.8507	0.0347	0.0051	10.2116	20.46	22.56	0.00	1.24	10.95
**G(S5)**	0.3360	0.2528	3.9013	1.2336	0.9294	0.0725	0.0019	10.6381	20.62	20.30	0.00	0.00	5.35

**G(S6)**	0.1312	0.0841	1.0401	0.3922	0.3858	0.0123	0.0004	3.7889	25.77	27.19	0.00	0.00	5.82
**G(S7)**	0.1164	0.0561	0.3380	0.1413	0.3220	0.0000	0.0006	1.9825	22.66	19.95	0.00	0.00	7.18
**H(S1)**	0.1896	0.1216	0.1326	0.2730	0.4724	0.0060	0.0074	3.3911	21.39	22.63	0.00	0.00	25.88
**H(S2)**	0.2795	0.2040	0.6287	0.5764	0.6562	0.0888	0.0478	6.1682	18.98	22.40	0.00	0.00	24.06
**H(S3)**	0.1758	0.1257	0.2196	0.3744	0.5273	0.0501	0.0061	4.0284	29.74	33.40	0.00	0.00	19.06
**H(S4)**	0.3364	0.1833	0.4615	1.1230	0.6583	0.0301	0.0045	6.3451	7.91	8.53	0.00	0.00	30.44
**H(S5)**	0.1500	0.2236	0.4296	0.7811	0.7415	0.0412	0.0350	6.0917	19.56	21.92	0.00	0.00	31.96
**H(S6)**	0.0179	0.0977	0.4259	0.3448	0.4150	0.0297	0.0015	2.8567	26.49	28.82	0.00	0.00	21.29
**H(S7)**	0.2725	0.1535	0.6136	0.7215	0.4945	0.0400	0.0294	5.0920	20.33	22.69	0.00	0.00	38.37
**I(S1)**	0.0655	0.0823	0.0416	0.1987	0.3123	0.6579	0.0015	2.7329	0.33	0.36	0.00	0.34	0.65
**I(S2)**	0.0090	0.0285	0.0228	0.2078	0.1660	0.0754	0.0006	1.1065	0.30	0.30	0.00	0.42	0.55
**I(S3)**	0.0038	0.0068	0.0191	0.0670	0.0730	0.0100	0.0003	0.4449	0.22	0.24	0.00	0.16	0.50
**I(S4)**	0.0453	0.0554	0.0393	0.4106	0.2540	0.0493	0.0021	1.8238	0.38	0.37	0.26	0.30	0.69
**I(S5)**	0.0295	0.0471	0.0374	0.2257	0.2405	0.2314	0.0008	1.7209	0.33	0.38	0.22	0.32	0.69
**I(S6)**	0.1892	0.2112	0.2268	1.8598	0.5190	0.6026	0.0069	7.0628	0.44	0.33	0.51	0.97	1.21
**I(S7)**	0.0068	0.0268	0.0411	0.0006	0.2560	0.6908	0.0005	1.9667	0.41	0.36	0.00	0.30	0.67

Note: TN= Total nucleosides, TA= Total amino acids

A was main root, B was lateral root, C was lateral root bark, D was main root bark, E was stem, F was leaf, G was pericarp, H was fruit pulp, I was seed kernels.

Wanlou 7 (S1), Wanlou 4-11 (S2), Wanlou 17 (S3), Wanlou 9 (S4), Wanlou 16 (S5), Wanlou 8 (S6), and Wanlou 7-8 (S7).

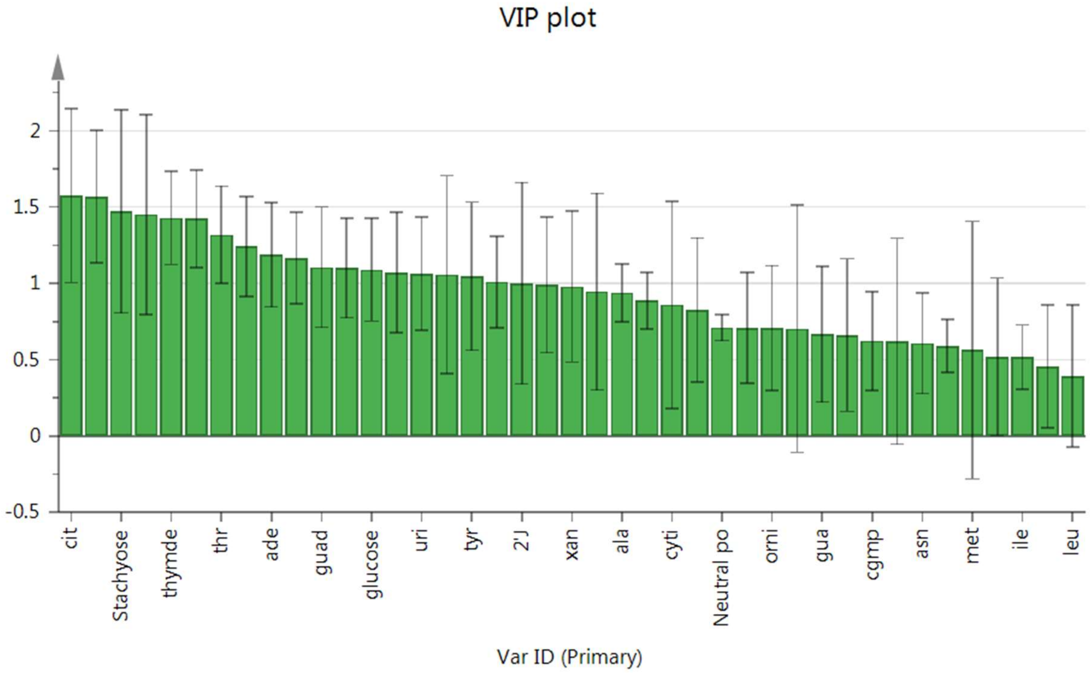

Figure S1.VIP plot of *T. kirilowii* samples.

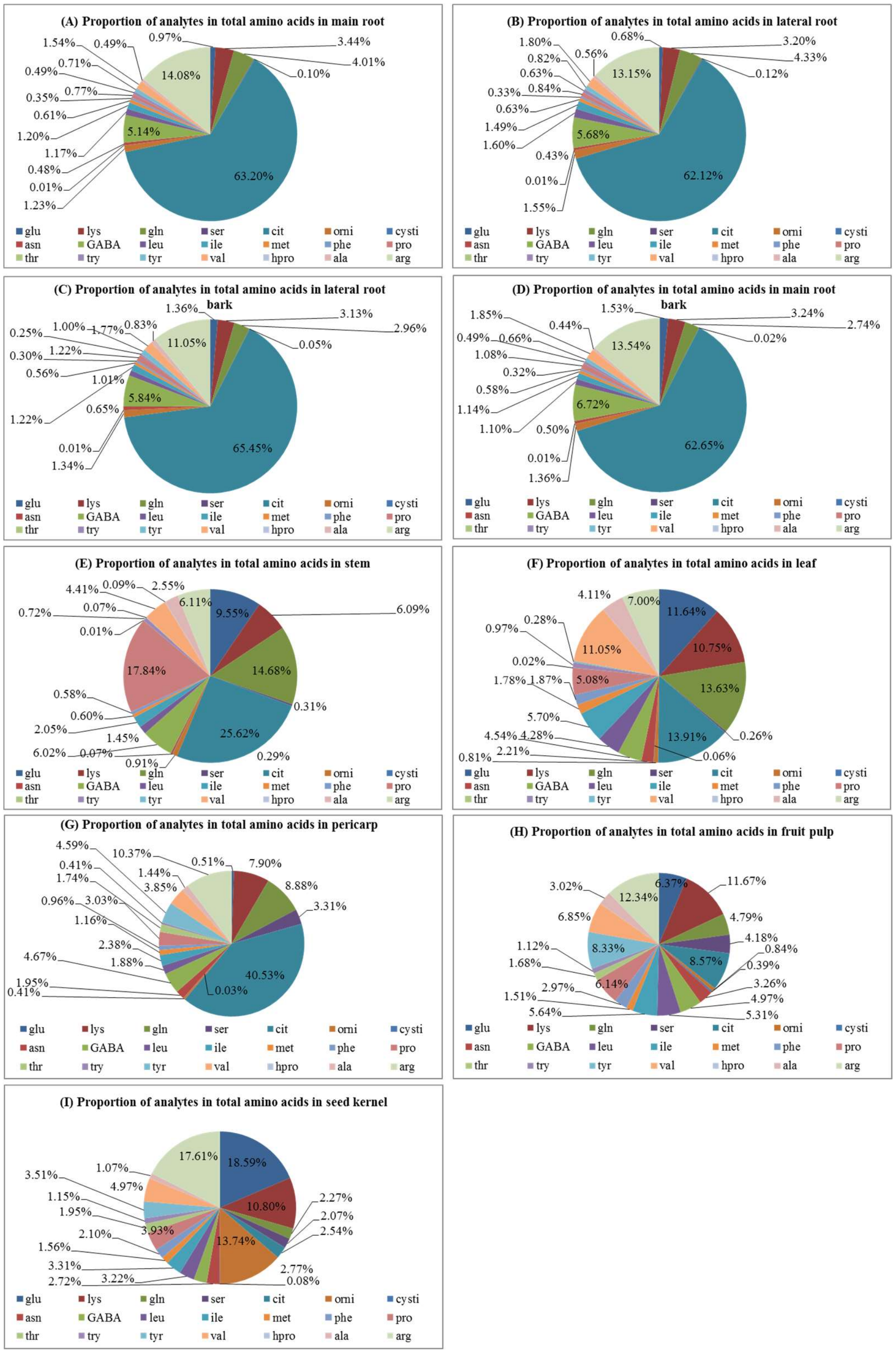

Figure S2. Proportion of the relatively abundant analytes in total amino acids.

Note: glu, L-glutamic acid; lys, L-lysine; gln, L-glutamine; ser, L-serine; cit, L-citrulline; orni, L-ornithine; cysti, L-cystine; asn, L-asparagine; GABA, γ-aminobutyric acid; leu, L-leucine; ile, iso-leucine; met, L-methionine; phe, L-phenylalanine; pro, L-proline; thr, L-threonine; try, L-tryptophan; tyr, L-tyrosine; val, L-valine; hprp, *trans*-4-hydroxy-L-proline; ala, L-alanine; arg, L-arginine

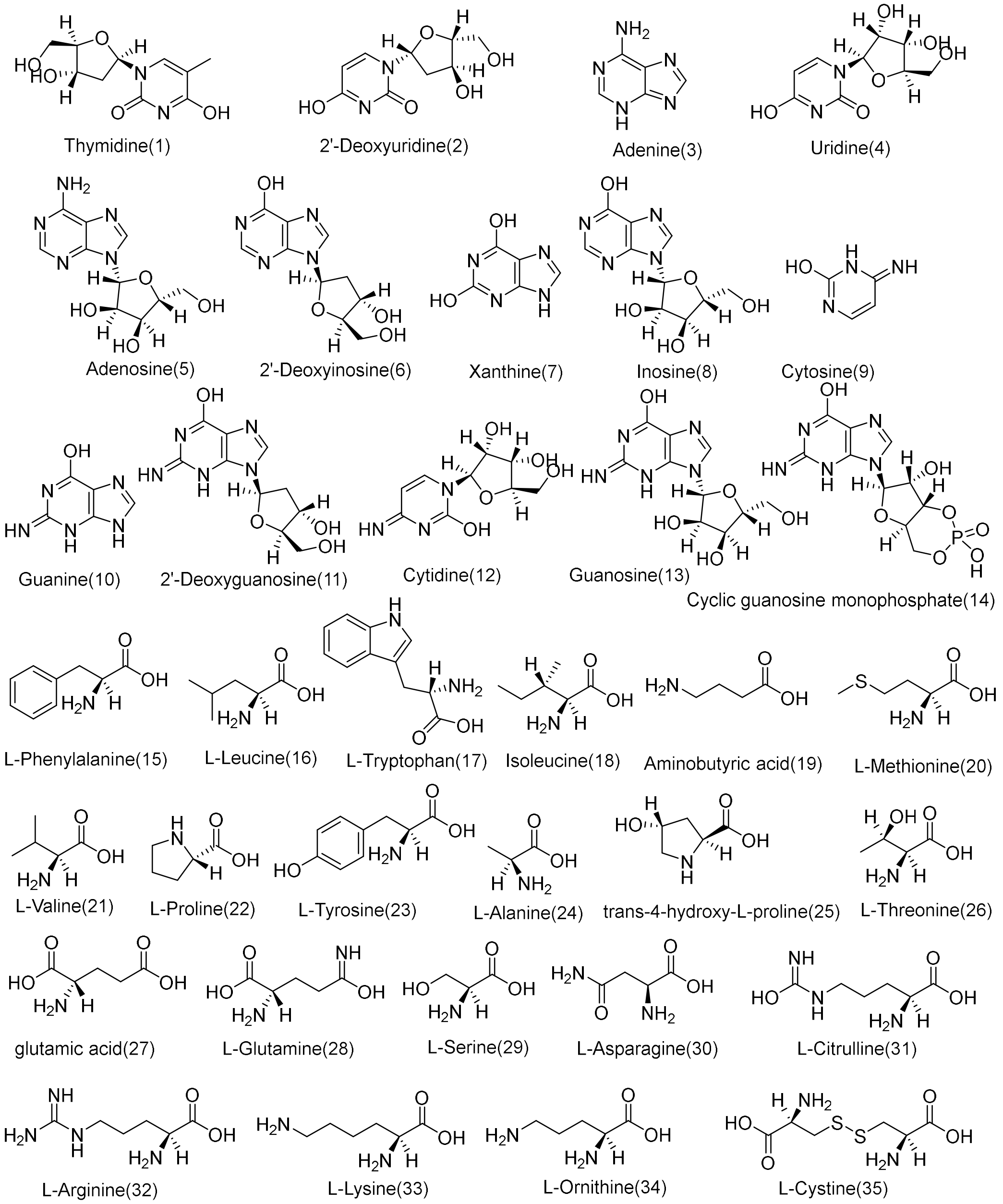

Figure S3. Chemical structures of investigated compounds
